# Supplementary material for: Realizing high-ranged thermoelectric performance in PbSnS2 crystals
Source: Nat Commun. 2022 Oct 8;13:5937. doi: 10.1038/s41467-022-33684-0 (PMC9547848; doi:10.1038/s41467-022-33684-0)
Supplement: Supplementary file 1 — Supplementary Information [file 41467_2022_33684_MOESM1_ESM.pdf]

Supplementary Information

Realizing high-ranged thermoelectric performance in $PbSnS_2$ crystals

Shaoping Zhan[1], Tao Hong[1], Bingchao Qin[1], Yingcai Zhu[1], Xiang Feng[1], Lizhong Su[1], Haonan Shi[1], Hao Liang[2], Qianfan Zhang[1], Xiang Gao[3], Zhen-Hua Ge[2], Lei Zheng[1], Dongyang Wang[4*] & Li-Dong Zhao[1,5*]

[1]School of Materials Science and Engineering, Beihang University, Beijing 100191, China

[2]Faculty of Materials Science and Engineering, Kunming University of Science and Technology, Kunming 650093, China

[3]Center for High Pressure Science and Technology Advanced Research (HPSTAR), Beijing 100094, China

[4]Henan Key Laboratory of Diamond Optoelectronic Materials and Devices, Key Laboratory of Material Physics, Ministry of Education, School of Physics, Zhengzhou University, Zhengzhou 450052, China

[5]Key Laboratory of Intelligent Sensing Materials and Chip Integration Technology of Zhejiang Province (2021E10022), Hangzhou Innovation Institute of Beihang University, Hangzhou 310051, China

[*]Corresponding author: wangdongyang@buaa.edu.cn; zhaolidong@buaa.edu.cn

Supplementary Methods

X-ray diffraction (XRD). Diffraction patterns of the powder or cleavage plane for undoped $PbSnS_2$ (carrier concentration is 3.6×10^{11} cm^{-3}) and Cl doped $PbSnS_2$ (carrier concentrations are 0.8×10^{19}, 1.3×10^{19} and 1.7×10^{19} cm^{-3}, respectively) crystals were recorded using D/max2200PC instrument with Cu $K\alpha$ (λ = 1.5418 Å) radiation in a reflection geometry on a diffractometer operating at 20 kV and 20 mA and equipped with a position-sensitive detector.

Differential scanning calorimetry (DSC) measurements. Undoped $PbSnS_2$ (carrier concentration is 3.6×10^{11} cm^{-3}) and Cl doped $PbSnS_2$ (carrier concentrations are 0.8×10^{19}, 1.3×10^{19} and 1.7×10^{19} cm^{-3}, respectively) crystals were cut into a cylinder with dimension of Φ ~5 mm and height of 3-5 mm to be tested (Setaram Labsys Evo-DSC 1600 °C, FRA). The obtained samples were added into an aluminum crucible while another crucible without sample as a blank, both heated up to ~843 K at a rate of 15 K min^{-1} under N_2 atmosphere. Similarly, the alumina standard sample were heated up under the same conditions. Therefore, the experimental heat capacity C_p of $PbSnS_2$ crystals could be acquired.

Hall measurements. The crystal samples to be measured were cut and polished into slices with size of ~6 mm × 6mm × 0.6mm. The temperature-dependent hall coefficient R_H was measured using the Van der Paw method on Lake Shore 8400 Series equipment at a reversible magnetic field of 0.9 T with a current of 10 mA. The measurement process from 300 to 773K was carried out in the Ar gas environment to prevent sample oxidation and protect the instrument. The Hall carrier concentration n_H and the Hall mobility μ_H can be obtained from formulae $n_H = 1/(eR_H)$ and $\mu_H = \sigma R_H$, where e and σ are electron charge and conductivity, respectively.

Bandgap measurements. The bulk crystals were ground into powder and passed through a 200-mesh sieve for room-temperature optical diffuse reflectance spectroscopy. The bandgap measurements were carried out using ultraviolet visible spectroscopy (Shimadzu Model UV-3600 Plus) and it is necessary to calibrate the instrument using $BaSO_4$ powder as a 100% reflection standard before measurements. The bandgap could be obtained by converting the reflectance (R) versus wavelength (λ) data to absorption data according to the Kubelka-Munk formula $\alpha/S = (1\text{-}R)^2/(2R)$ and formula $E = 1240/\lambda$, where α, S, and E represent absorption coefficient, scattering coefficient and energy, respectively.

Single parabolic band (SPB) model. The Pisarenko relation is calculated by the SPB model according to equations **S1-4**:

$$S = \frac{k_B}{e}\left[\frac{2F_1(\delta)}{F_0(\delta)} - \delta\right] \tag{S1}$$

$$F_x(\delta) = \int_0^\infty \frac{\varepsilon^x}{1+\exp(\varepsilon-\delta)} d\varepsilon \tag{S2}$$

$$n_H = \frac{(2m^* k_B T)^{3/2}}{2\pi^2\hbar^3} \frac{F_{1/2}(\delta)}{r_H} \tag{S3}$$

$$r_H = \frac{3}{4}\frac{F_{1/2}(\delta)F_{-1/2}(\delta)}{[F_0(\delta)]^2} \tag{S4}$$

where k_B is the Boltzmann constant, e is the electron charge, δ is the reduced Fermi energy, $F_x(\delta)$ is Fermi integral, n_H is carrier concentration, m^* is the density of state (DOS) effective mass, T is the temperature, $\hbar$ is the reduced Planck's constant and r_H is the Hall coefficient.

Weighted mobility. Based on the SPB model and measured electrical conductivity (σ) and Seebeck coefficient (S), weight mobility (μ_W) can be obtained according to equations **S2** and **S5-6**:

$$\mu_W = \frac{3\sigma}{8\pi e F_0(\delta)}\left(\frac{h^2}{2m_e k_B T}\right)^{3/2} \tag{S5}$$

$$S = \pm\frac{k_B}{e}\left[\frac{(r+5/2)F_{r+3/2}(\delta)}{(r+3/2)F_{r+1/2}(\delta)} - \delta\right] \tag{S6}$$

where h is Planck's constant, m_e is unit mass of free electron and r is the scattering factor and equals -1/2 due to assuming that the carrier scattering is mainly caused by acoustic phonons.

Debye model. Considering the contribution of phonon and lattice thermal expansion, the total heat capacity $C_{p,tot}(T)$ can be expressed as[1]:

$$C_{p,tot}(T) = C_{p,ph}(T) + C_{p,D}(T) \tag{S7}$$

where $C_{p,ph}(T)$ and $C_{p,D}(T)$ donate the contributions of phonon and lattice dilation to the $C_{p,tot}(T)$, respectively. Based on the elastic wave approximation and considering the effects of thermal expansion to the heat capacity, $C_{p,ph}(T)$ and $C_{p,D}(T)$ can be expressed as:

$$C_{p,ph}(T/\theta_D) = 9R\left(\frac{T}{\theta_D}\right)^3 \int_0^{\theta_D} \frac{x^4 e^x}{(e^x-1)^2} dx \tag{S8}$$

$$C_{p,D}(T) = C_{ele,D}(T) + C_{ph,D}(T) = \frac{9BT\alpha^2}{10^6\rho} \tag{S9}$$

where θ_D is the Debye temperature, R is the gas constant (8.314 J mol^{-1} K^{-1}), and $x = \hbar\omega/k_B T$, in which ω is phonon vibration frequency, B is the isothermal bulk modulus, α is the linear coefficient of thermal expansion and ρ is the sample density, respectively.

Calculation for the Lorenz number. Compared with rigorous single non-parabolic band and multiple bands model calculation, there is a more precise way to estimate Lorenz number L with the error is within 10% based on SPB model. The L can be obtained according to equations **S2** and **S10-12**[2]:

$$L=\left(\frac{k_B}{e}\right)^2\left\{\frac{(r+7/2)F_{r+5/2}(\delta)}{(r+3/2)F_{r+1/2}(\delta)}-\left[\frac{(r+5/2)F_{r+3/2}(\delta)}{(r+3/2)F_{r+1/2}(\delta)}\right]^2\right\} \tag{S10}$$

$$S=\frac{k_B}{e}\left[\frac{(r+5/2)F_{r+3/2}(\delta)}{(r+3/2)F_{r+1/2}(\delta)}-\delta\right] \tag{S11}$$

$$\delta=\frac{E_f}{k_B T} \tag{S12}$$

where r is the scattering factor and equals -1/2 due to assuming that the carrier scattering is mainly caused by acoustic phonons and S is measured Seebeck coefficient, E_f is the Fermi energy, respectively.

Calculation of average *ZT* (*ZT*ave). ZT_{ave} can be estimated through the following equations[3,4]:

$$ZT_{ave}=\frac{1}{T_h-T_c}\int_{T_c}^{T_h} ZTdT \tag{S13}$$

where T_h and T_c are the hot and cold side temperature, respectively.

X-ray absorption fine structure (XAFS) spectroscopy. The XAFS of Pb L_3-edge and Sn K-edge for $PbSnS_2$ were measured in transmission mode at BL01B1 beamline of Spring-8. The storage ring runs in top-up mode with a 99.5 mA accumulated current during the measurements. A Si (311) double-crystal monochromator was employed to tune the energy of X-ray beam. All experimental XAFS spectra were preprocessed using the IFFEFIT package[5]. We calculated the X-ray absorption near edge structure (XANES) of Pb L_3-edge and Sn K-edge for $PbSnS_2$ using four possible crystal structure. These calculations were conducted based on the full multiple scattering (FMS) theory using FEFF9 program[6]. The atomic scattering potential was estimated by the self-consistent field (SCF) method. The cluster radius for SCF and FMS was fixed as 8 and 10 Angstrom, respectively, to get a good convergence.

Density functional theory calculation. First-principle simulations were performed in the framework of density functional theory (DFT) as implemented in the Vienna Ab initio Simulation Package (VASP)[7,8]. The electron exchange and correlation effects were treated using the generalized gradient approximation (GGA) described by the Perdew-Burke-Ernzerhof (PBE) formulation[9]. The Gaussian smearing method was used to describe the Fermi-Dirac distribution function, and the width of the smearing was set to 0.01 eV. The energy cutoff for plane-wave expansion of the projector augmented waves (PAWs)[10,11] was set to 500 eV. For the relaxation of the crystal structures, the total energy convergence criteria and the atomic force convergence criteria was set to 10^{-6} eV and 10^{-2} eV Å^{-1}, respectively. The Gamma-centered Monkhorst-Pack method was used for the sampling of the Brillouin zone[12]. 15 × 14 × 5, 7 × 14 × 5, 7 × 7 × 5, 7 ×7 × 3, 4 × 7 ×5 and 4 × 7

× 3 k-point meshes were adopted by 1 × 1 × 1, 2 × 1 × 1, 2 × 2× 1, 2 × 2 × 2, 4 × 2 × 1 and 4 × 2 × 2 supercells, respectively. In Model 0, Pb and Sn atoms are randomly distributed over the metal sublattice. To evaluate the energy of Model 0, for each supercell, we randomly selected 4 distributions of Pb and Sn atoms, and regarded the average energy of these 4 structures as the predicted energy of this supercell. The calculation of the XRD pattern was performed using the Python package Pymatgen[13]. The wave length of the X-ray was set to 0.6887 Å. To evaluate the formation energy of the Cl-doped system, a S atom was substituted by Cl in the 2 × 1 × 1, 2 × 2 × 1, 2 × 2 × 2, and 4 × 2 × 2 supercells, representing $Pb_4Sn_4S_7Cl$, $Pb_8Sn_8S_{15}Cl$, $Pb_{16}Sn_{16}S_{31}Cl$, and $Pb_{32}Sn_{32}S_{63}Cl$, respectively. The formation energy E_f of $Pb_aSn_bS_cCl_d$ is expressed as:

$$E_{\mathrm{f}} = \frac{E_{\mathrm{tot}} - (aE_{\mathrm{Pb}} + bE_{\mathrm{Sn}} + cE_{\mathrm{S}} + dE_{\mathrm{Cl}})}{a + b + c + d}$$

where E_{tot} is the total energy of $Pb_aSn_bS_cCl_d$, and E_{Pb}, E_{Sn}, E_S, and E_{Cl} are the total energy per atom of Pb, Sn, S, and Cl in their ground state, which were obtained from the Materials Project (MP) database[14] with material ID of mp-20483, mp-117, mp-96, and mp-1008394, respectively. Since structures from MP were adopted, inputs of MPRelaxSet, MPStaticSet, and MaterialsProject2020Compatibility implemented in Pymatgen were used for consistency.

Electronic band structure calculation. The experimental structures at elevated temperatures (from 300 to 823 K) deriving from the SR-XRD data (**Table. S1-S2**). The calculated band structures of the optimal Cl doped $PbSnS_2$ at elevated temperature were presented in **Fig. S8**. The valence band maximum (VBM) and conduction band maximum (CBM) are located at Y point and along Γ-X directions, respectively. Only the high symmetric path in the Brillouin zone was adopted to plot the electronic band structures, it clearly shows very strong multi-valley character, especially the lower energy CBM2 along Γ-Y direction and CBM3 around Γ points, respectively. The energy difference between VBM1 and VBM2 gradually decreases, while the energy offset between VBM1 and VBM3 decrease first and then increase with increasing temperature. In other word, VBM1 and VBM3 become closer from 300 to ~ 623 K and experience band convergence around 623 K, above that, the two bands separate. The effective mass decreases in the entire temperature range, which is favorable for higher carrier mobility and electrical conductivity.

Phonon dispersion calculation. The finite displacement method was adopted to calculate the harmonic interatomic force constant using the Phonopy[15] code with 4 × 4 × 1 (128 atoms) supercells. The Grüneisen parameters provide an estimation of the anharmonic strength in a compound. In order to calculate the mode Grüneisen parameters, the quasi-harmonic approximation

(QHA) was adopted and the system volume isotropically compressed by 2 % from the relaxed equilibrium volume. The mode Grüneisen parameter $\gamma(\boldsymbol{q}, v)$ at the wave vector $\boldsymbol{q}$ and band index i can be expressed as:

$$\gamma(\boldsymbol{q}, i) = -\frac{V_0}{\omega(\boldsymbol{q}, i)} \times \frac{\partial \omega(\boldsymbol{q}, i)}{\partial V} \tag{S15}$$

where V_0 is the equilibrium volume and $\omega(\boldsymbol{q}, i)$ is the mode phonon frequency.

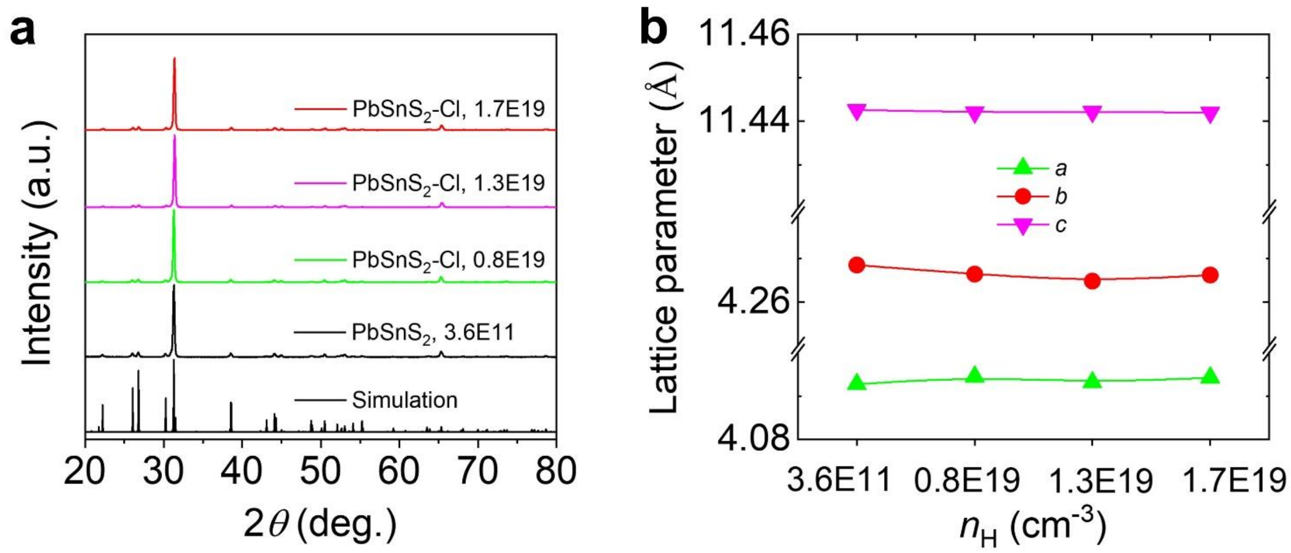

Supplementary Figure 1. (a) Room-temperature powder X-ray diffraction patterns. (b) Refined lattice parameters along different crystal directions for undoped and Cl doped $PbSnS_2$.

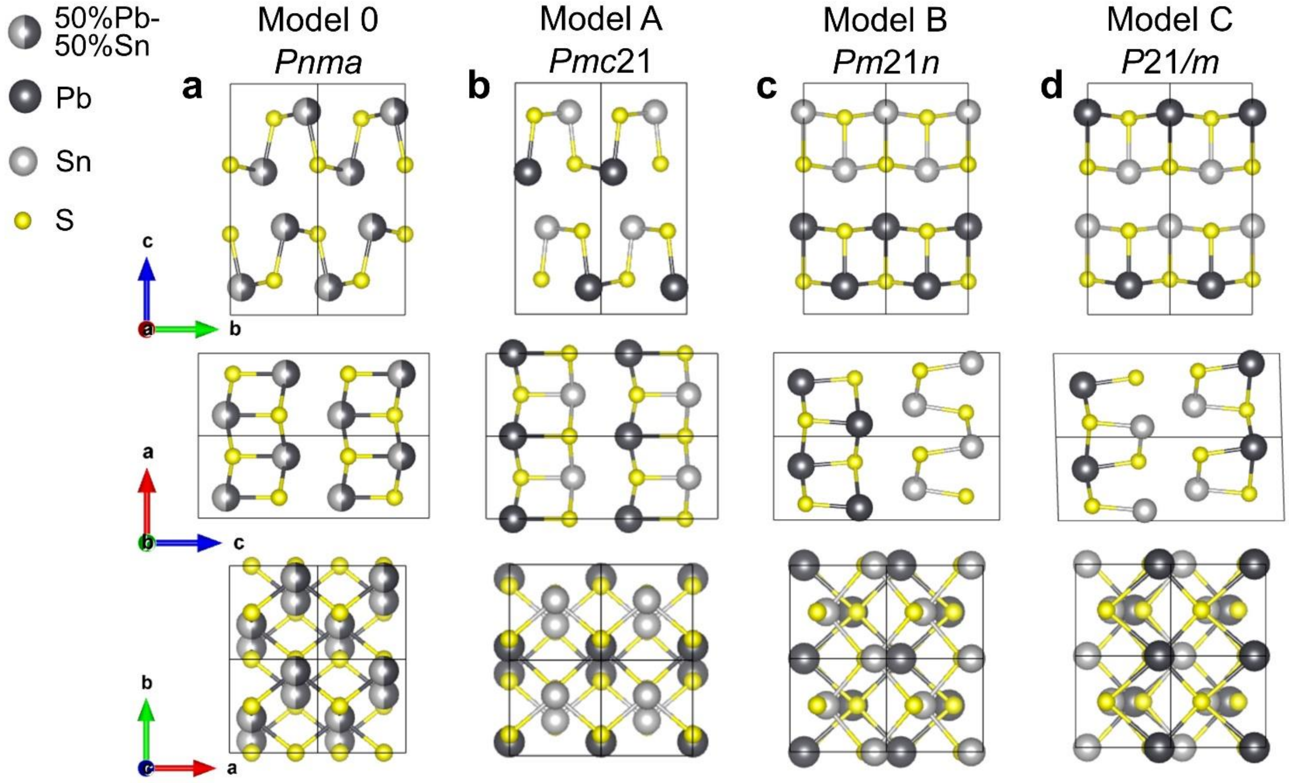

Supplementary Figure 2. Four models of $PbSnS_2$ summarized in the literature[16].

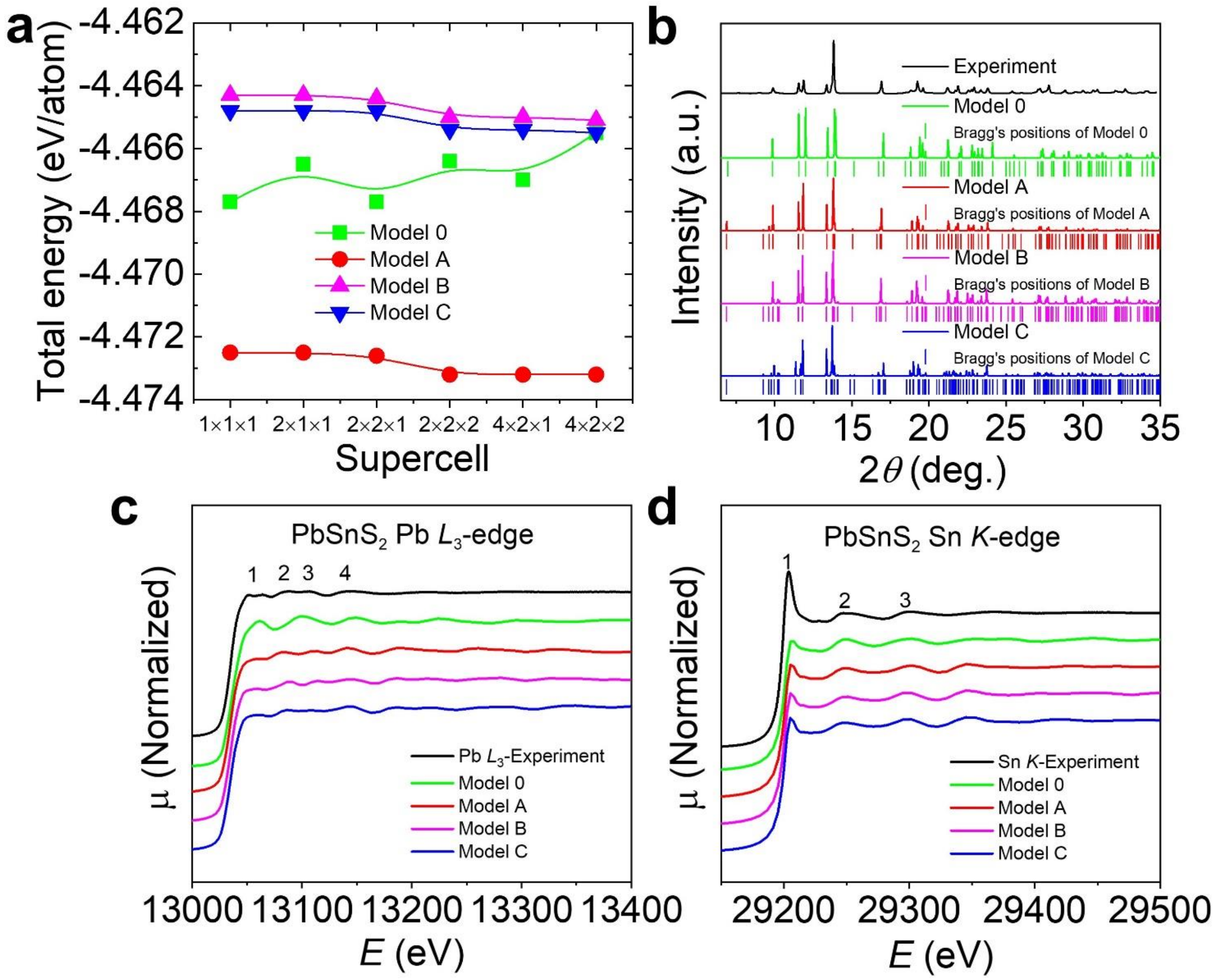

Supplementary Figure 3. Total energy and comparison of relative calculation with experimental data for four models. (a) Total energy. Comparison of simulated (b) diffraction peaks, (c) Pb L_3-edge and (d) Sn *K*-edge with relative experimental data.

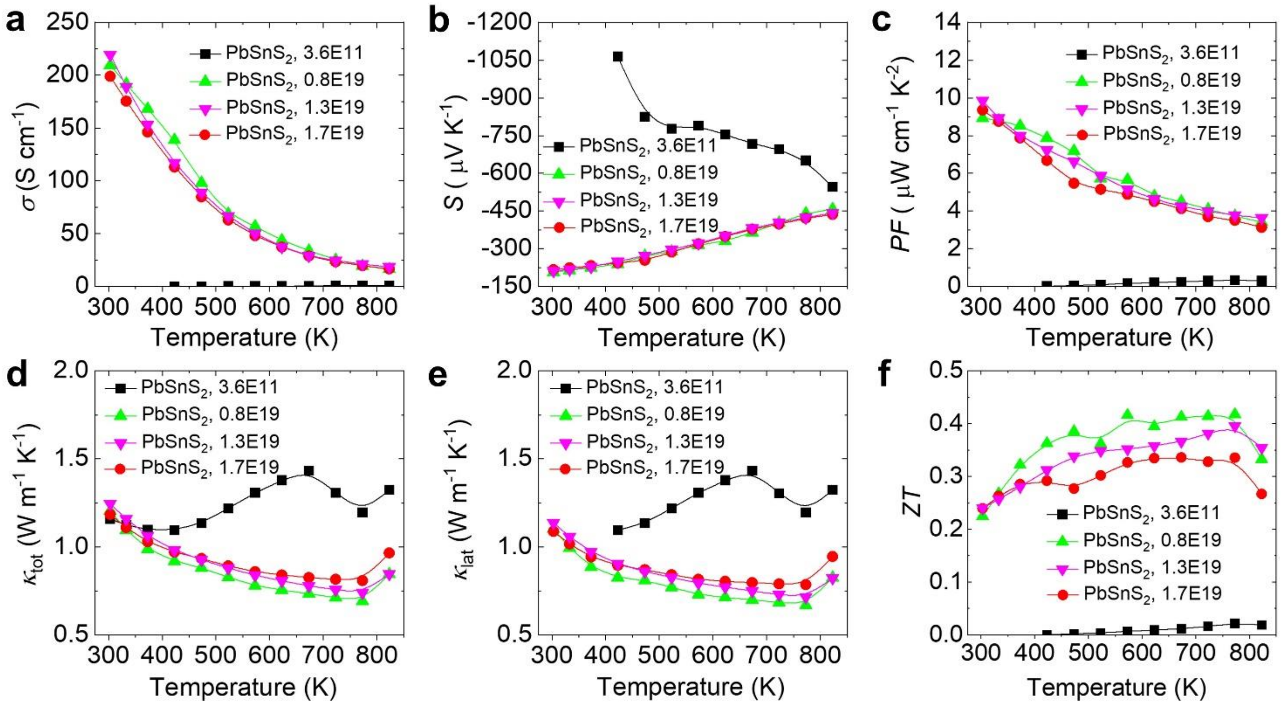

Supplementary Figure 4. Temperature-dependent thermoelectric performance of undoped and Cl doped $PbSnS_2$ crystals along in-plane direction. (a) Electrical conductivity. (b) Seebeck coefficient. (c) Power factor. (d) Total thermal conductivity. (e) Lattice thermal conductivity and (f) *ZT* values.

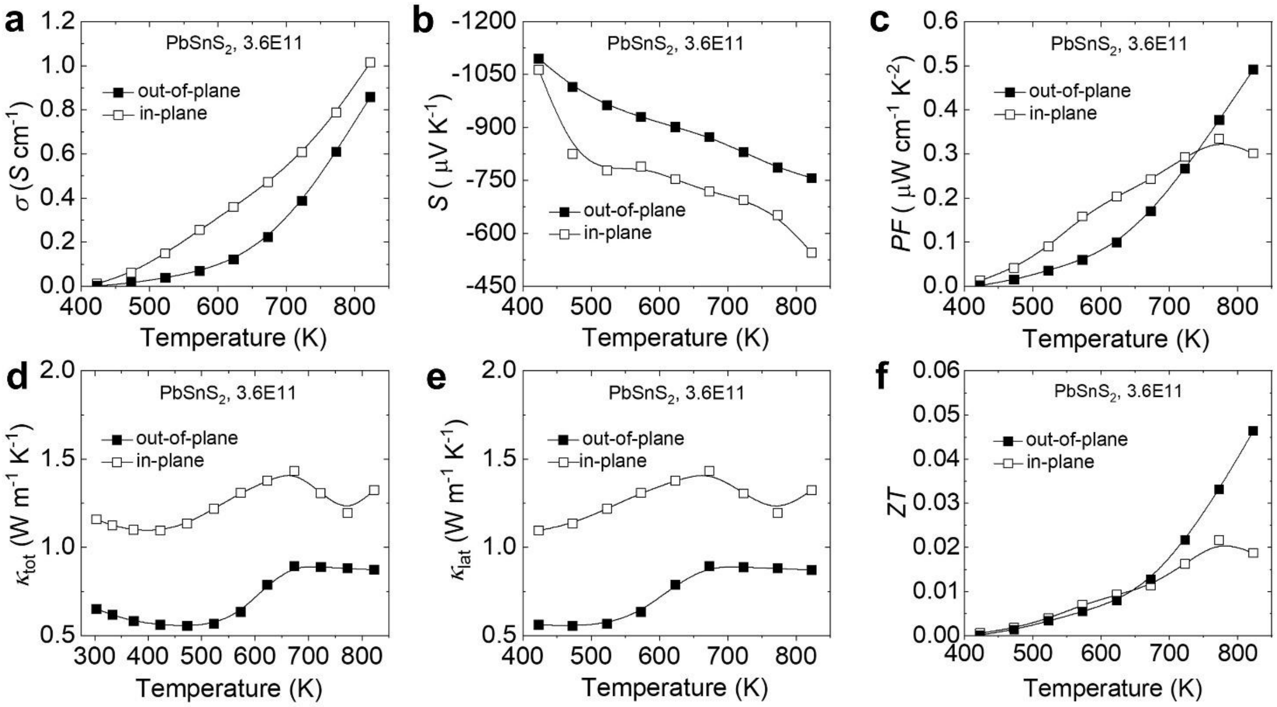

Supplementary Figure 5. Comparison of thermoelectric performance for undoped $PbSnS_2$ crystal along out-of-plane and in-plane directions. (a) Electrical conductivity. (b) Seebeck coefficient. (c) Power factor. (d) Total thermal conductivity. (e) Lattice thermal conductivity and (f) *ZT* values.

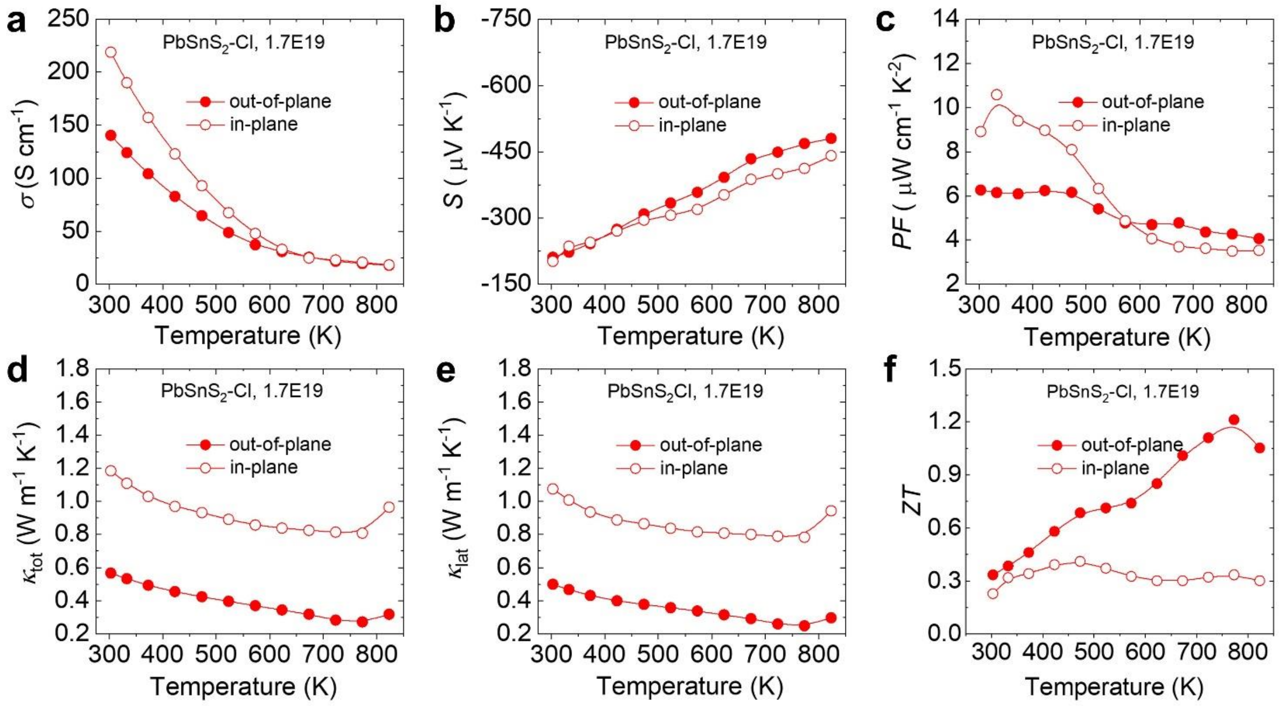

Supplementary Figure 6. Comparison of thermoelectric performance for the optimal Cl doped $PbSnS_2$ crystal along out-of-plane and in-plane directions. (a) Electrical conductivity. (b) Seebeck coefficient. (c) Power factor. (d) Total thermal conductivity. (e) Lattice thermal conductivity and (f) *ZT* values.

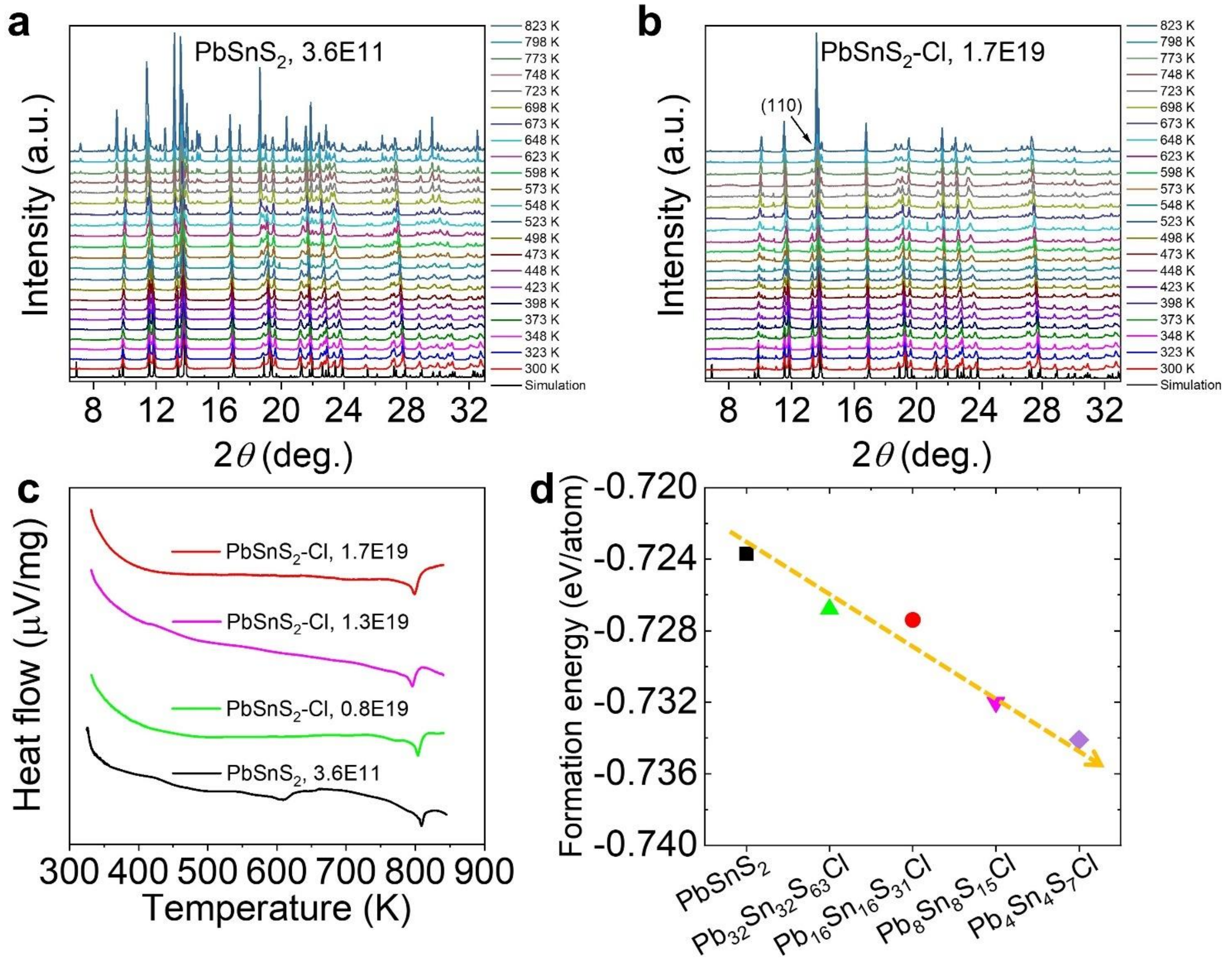

Supplementary Figure 7. High temperature SR-XRD data of (a) undoped $PbSnS_2$ and (b) the optimal Cl doped $PbSnS_2$. $PbCl_2$ phase can be indexed within 300-723 K in the optimal Cl doped $PbSnS_2$, indicating the exceeding solid solubility of $PbCl_2$ in $PbSnS_2$ matrix. (c) DSC measurements of undoped and Cl doped $PbSnS_2$. Undoped $PbSnS_2$ might start to partially decompose into PbS and SnS at ~ 623 K, and the structural phase transition temperature of all samples are in the temperature range of 773-823 K, which are consistent with the SR-XRD data. (d) The comparison of the formation energy between undoped and Cl doped $PbSnS_2$ with different compositions. Phase stability of $PbSnS_2$ was enhanced due to lower formation energy after Cl doping.

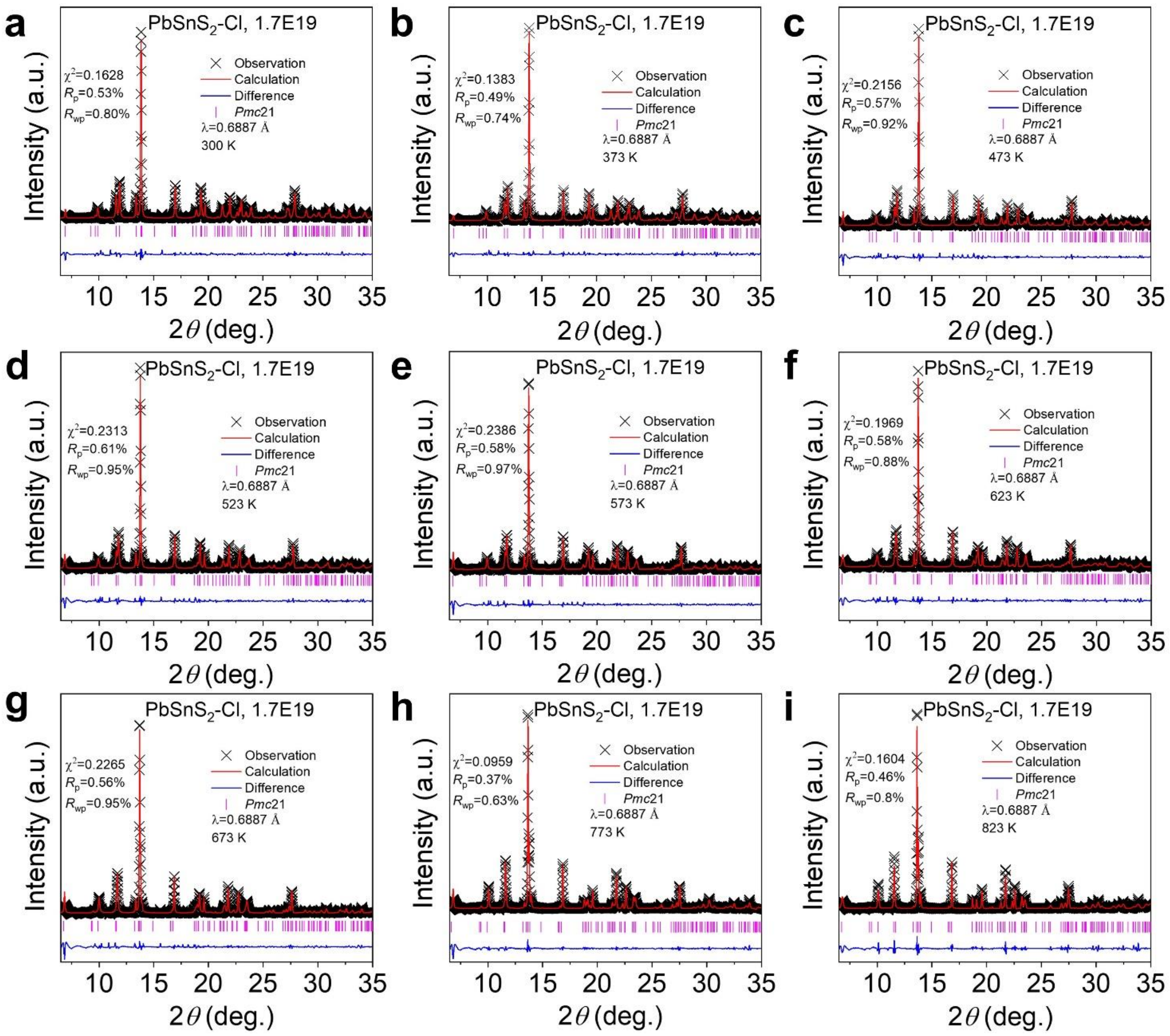

Supplementary Figure 8. Rietveld refinements for the optimal Cl doped $PbSnS_2$ sample at various temperature: (a) 300 K, (b) 373 K, (c) 473 K, (d) 523 K, (e) 573 K, (f) 623 K, (g) 673 K, (h) 773 K and (i) 823 K.

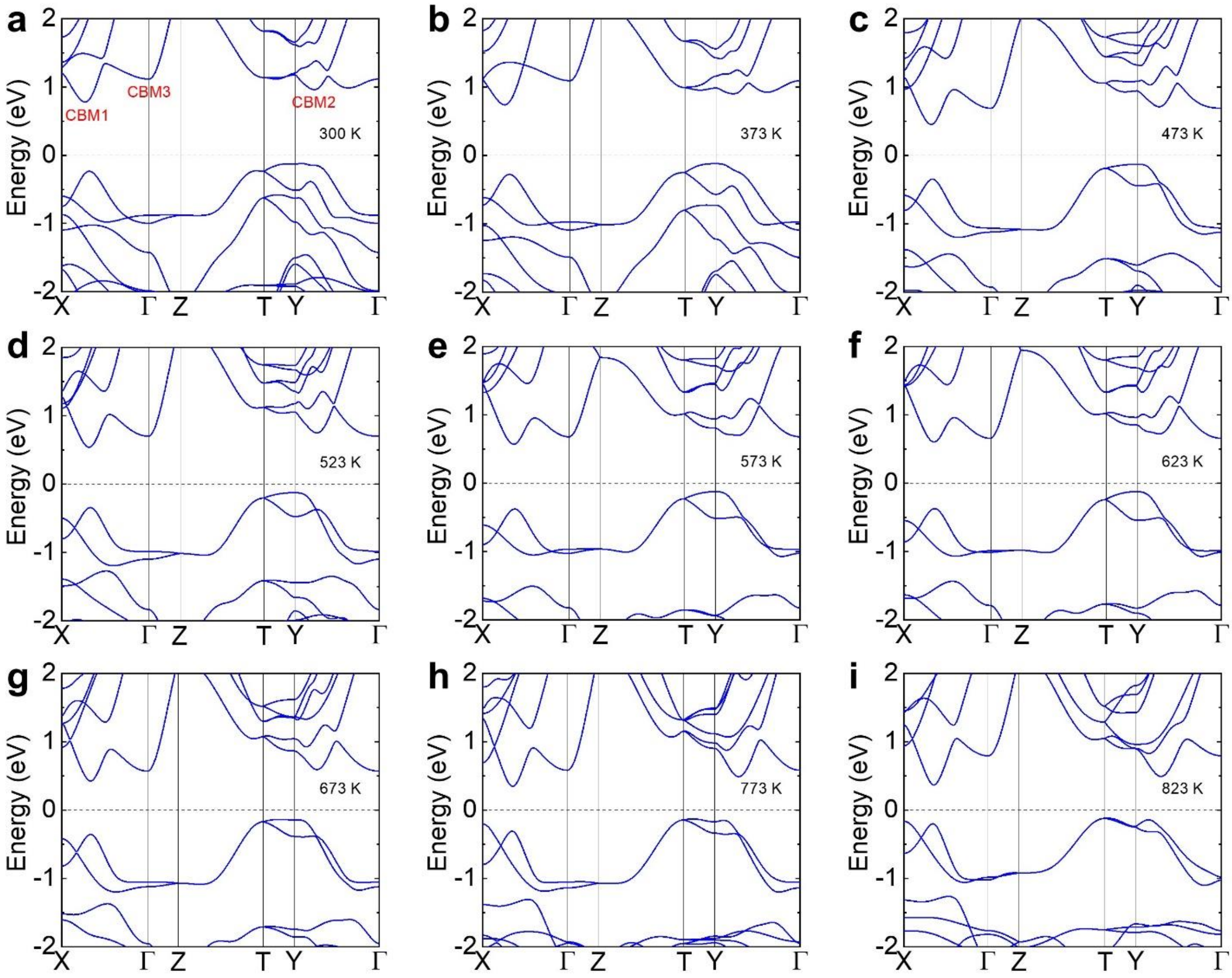

Supplementary Figure 9. The electronic band structures of the optimal Cl doped $PbSnS_2$ sample at various temperature: (a) 300 K, (b) 373 K, (c) 473 K, (d) 523 K, (e) 573 K, (f) 623 K, (g) 673 K, (h) 773 K and (i) 823 K.

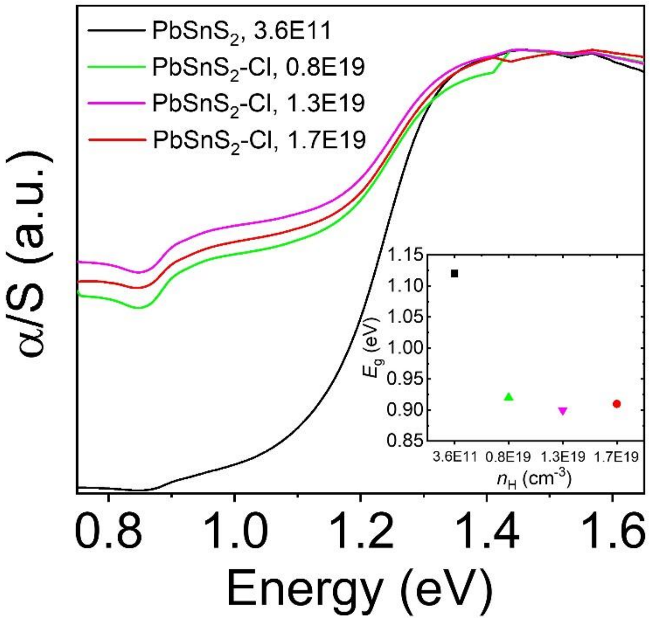

Supplementary Figure 10. Bandgap of undoped and Cl doped $PbSnS_2$ samples at room temperature.

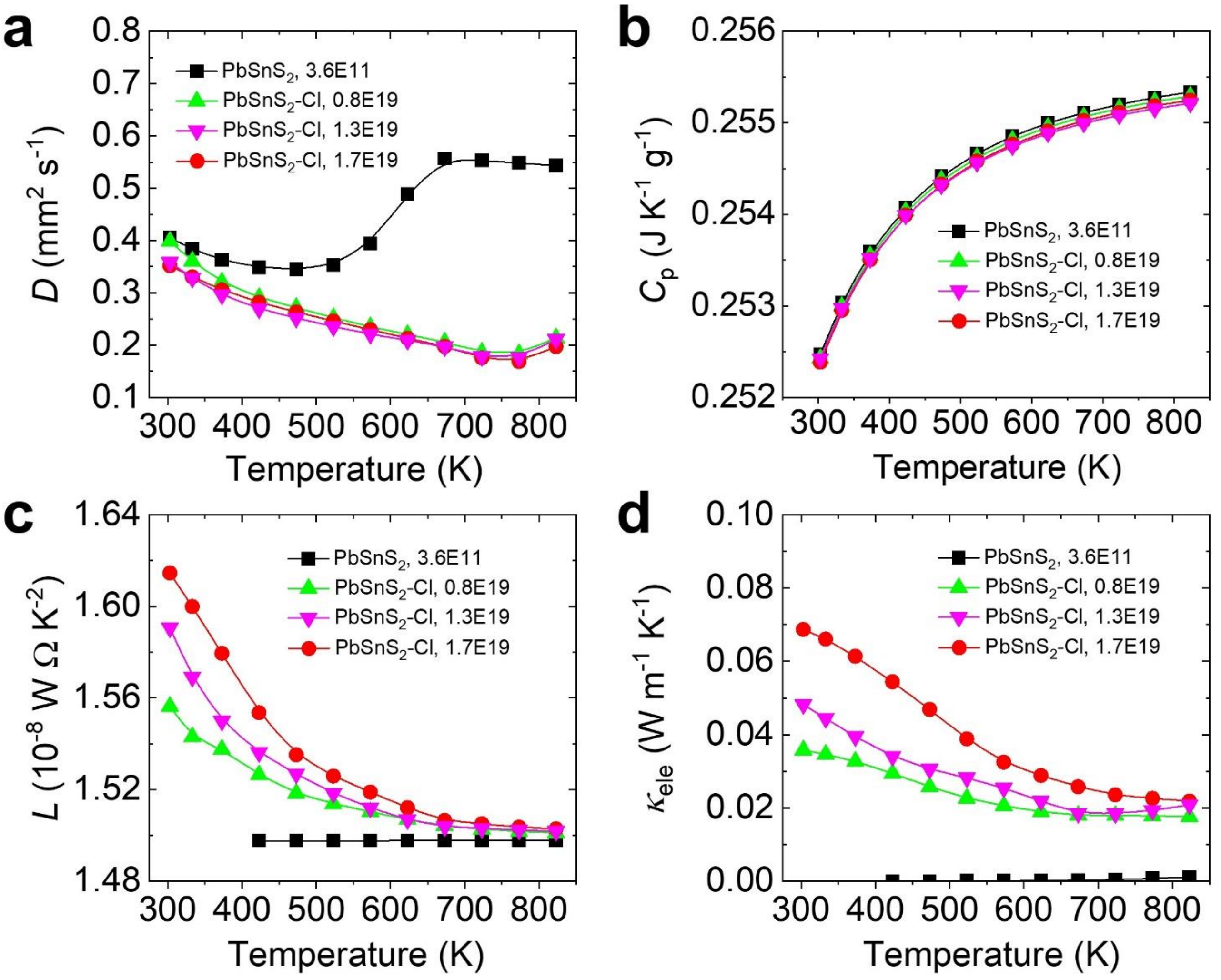

Supplementary Figure 11. Temperature-dependent thermoelectric performance for undoped and Cl doped $PbSnS_2$ crystals along out-of-plane direction. (a) Thermal diffusivity. (b) Heat capacity calculated using Debye model. (c) Lorenz number and (d) electronic thermal conductivity.

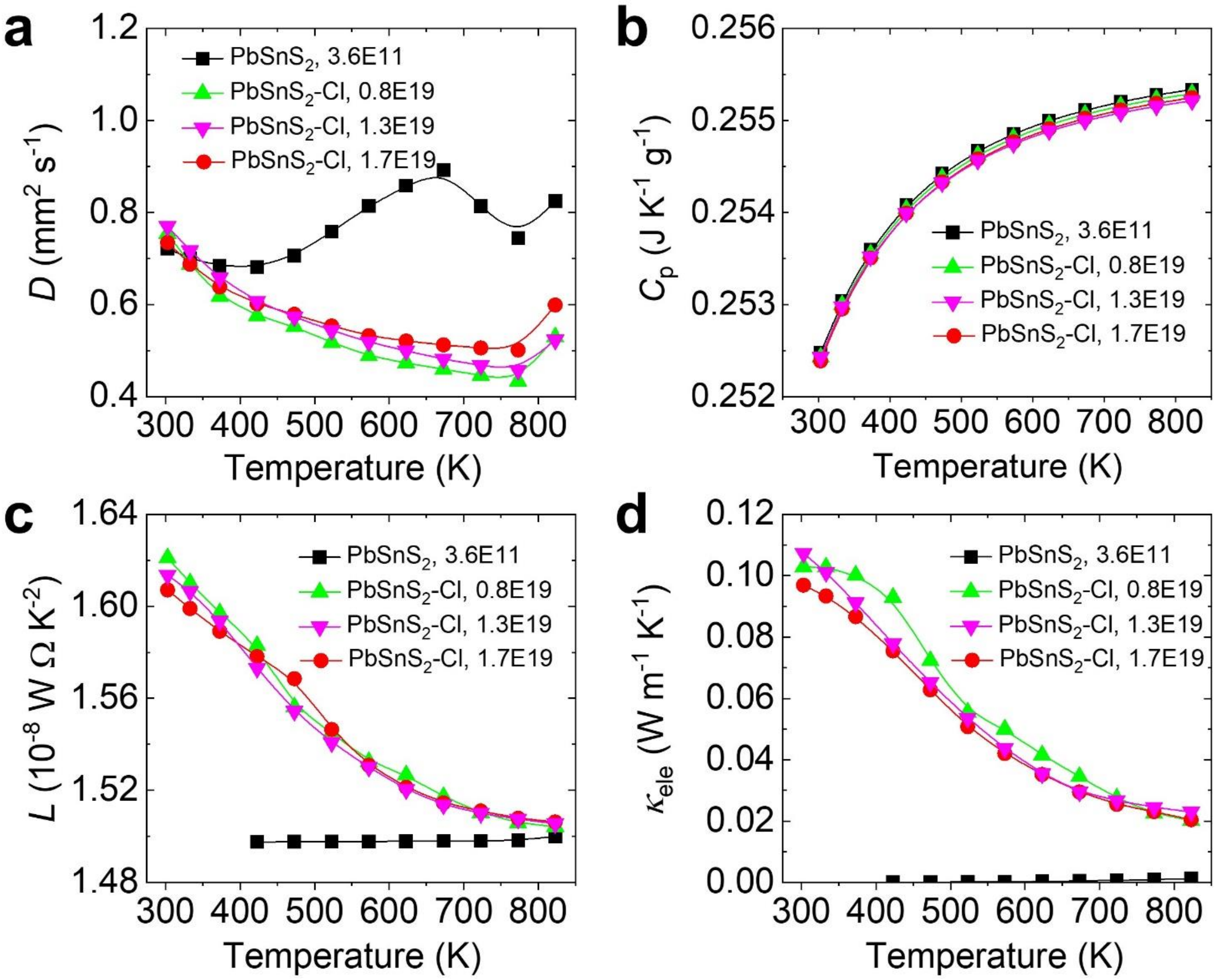

Supplementary Figure 12. Temperature-dependent thermoelectric performance for undoped and Cl doped $PbSnS_2$ crystals along in-plane direction. (a) Thermal diffusivity. (b) Heat capacity calculated using Debye model. (c) Lorenz number and (d) electronic thermal conductivity.

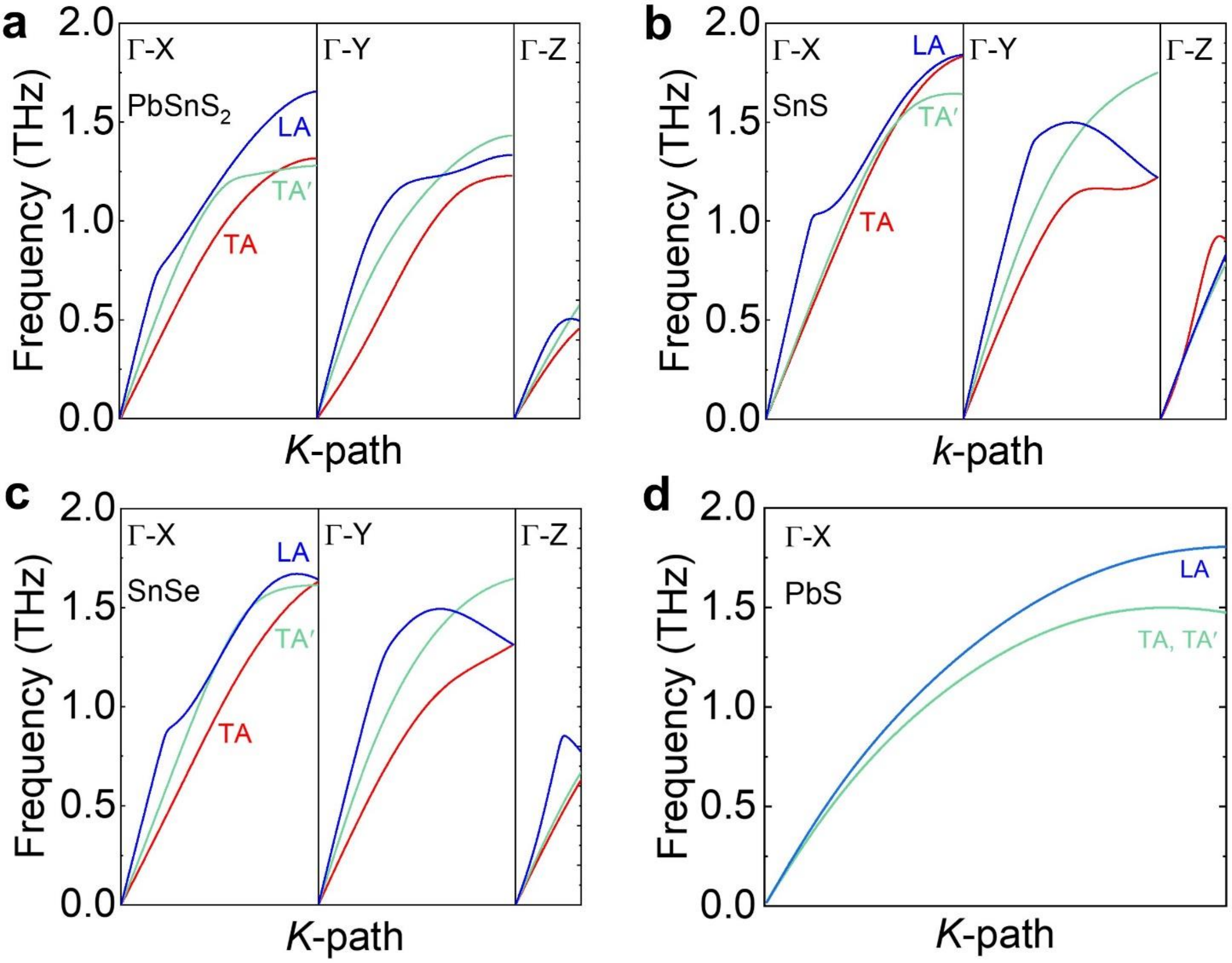

Supplementary Figure 13. Acoustic phonon modes for (a) $PbSnS_2$, (b) SnS, (c) SnSe and (d) PbS.

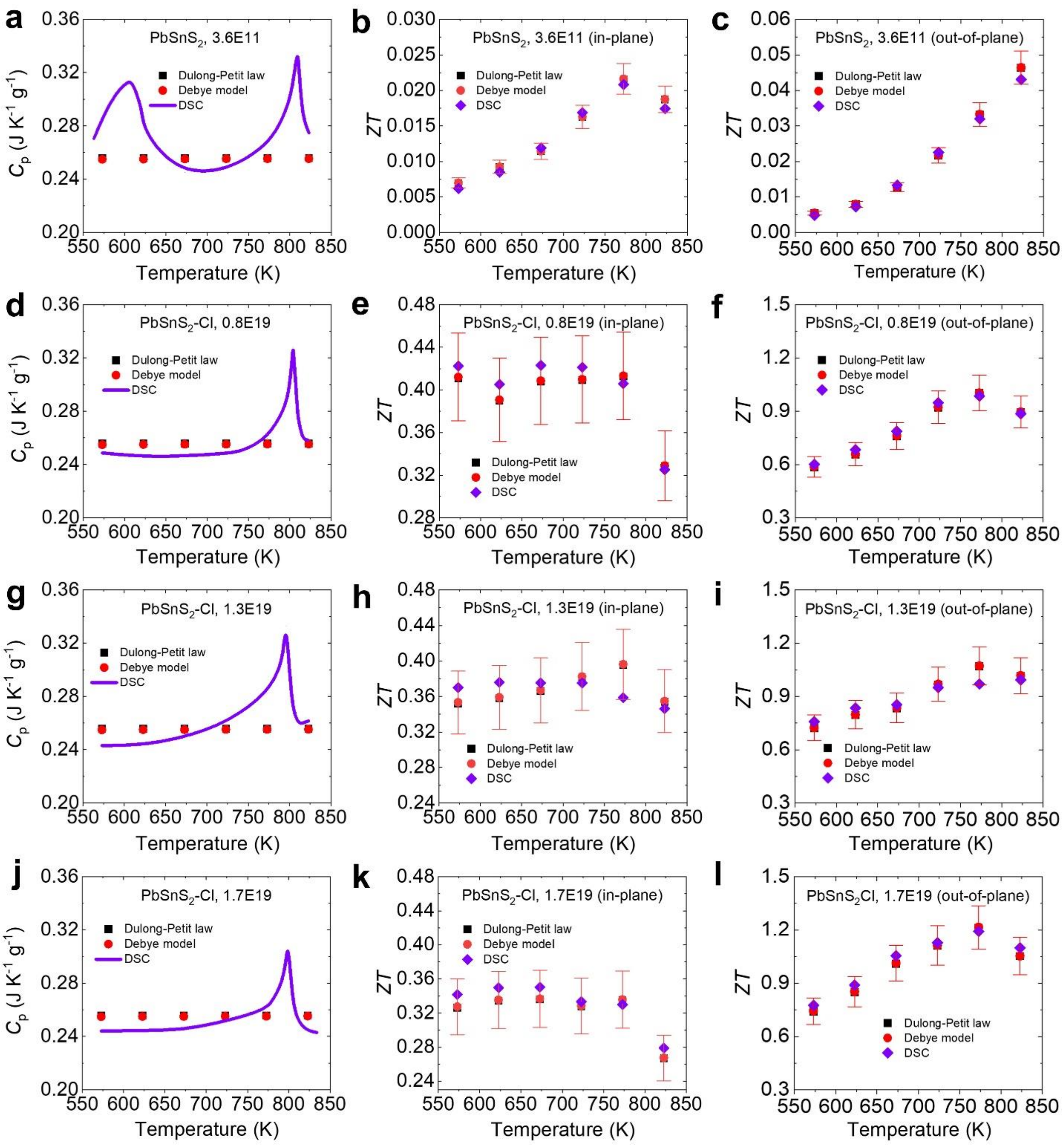

Supplementary Figure 14. (a, d, g and j) Temperature-dependent heat capacity of undoped and Cl doped $PbSnS_2$ obtained from different methods, including Dulong-Petti law, Debye model and experimental DSC data. (b, e, h and k) Temperature-dependent *ZT* values along in-plane direction of undoped and Cl doped $PbSnS_2$ calculated according to different heat capacities. (c, f, i and l) Temperature-dependent *ZT* values along out-of-plane direction of undoped and Cl doped $PbSnS_2$ calculated according to different heat capacities. The results of these three different methods showed a good consistency within the 20% error range. Error bars are ± 10%.

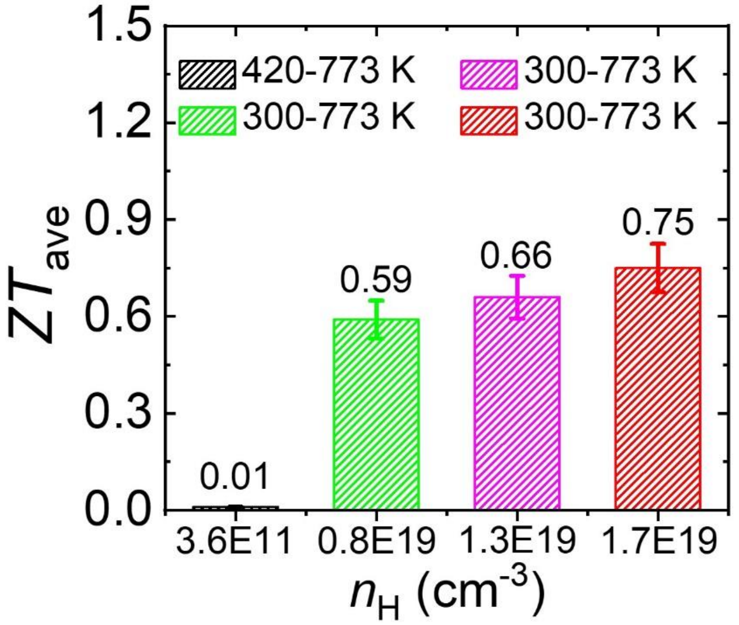

Supplementary Figure 15. Average *ZT* values for undoped $PbSnS_2$ (3.6×10^{11} cm^{-3}) and Cl doped $PbSnS_2$ (0.8×10^{19}, 1.3×10^{19}, 1.7×10^{19} cm^{-3}). Error bars are ± 10%.

Supplementary Table 1. Temperature-dependent lattice parameters for the optimal Cl doped $PbSnS_2$ sample.

Temperature (K)	*a* (Å)	*b* (Å)	*c* (Å)
300	4.0936	4.2719	11.4334
373	4.1083	4.2639	11.4541
473	4.1209	4.2467	11.4777
523	4.1318	4.2383	11.4903
573	4.1462	4.2272	11.5100
623	4.1575	4.2133	11.5195
673	4.1734	4.2058	11.5432
773	4.2092	4.1686	11.5724
823	4.2350	4.1552	11.5985

Supplementary Table 2. Temperature-dependent atomic positions for the optimal Cl doped $PbSnS_2$ sample.

Temperature (K)	Pb			Sn			S1			S2		
	x	*y*	*z*	*x*	*y*	*z*	*x*	*y*	*z*	*x*	*y*	*z*
300	0	0.1492	0.6259	0.5	0.3839	0.3851	0	0.2170	0.8694	0.5	0.2530	0.1471
373	0	0.1550	0.6213	0.5	0.3783	0.3783	0	0.2420	0.8708	0.5	0.2340	0.1441
473	0	0.1509	0.6040	0.5	0.3629	0.3601	0	0.2620	0.8599	0.5	0.3140	0.1533
523	0	0.1486	0.6061	0.5	0.3604	0.3622	0	0.2310	0.8582	0.5	0.2970	0.1534
573	0	0.1614	0.5957	0.5	0.3564	0.3528	0	0.2770	0.8518	0.5	0.2680	0.1595
623	0	0.1627	0.5959	0.5	0.3462	0.3523	0	0.2770	0.8509	0.5	0.2590	0.1553
673	0	0.1784	0.5987	0.5	0.3516	0.3551	0	0.2260	0.8564	0.5	0.2700	0.1569
773	0	0.1981	0.5943	0.5	0.3030	0.3519	0	0.2100	0.8525	0.5	0.2660	0.1660
823	0	0.2340	0.5816	0.5	0.2840	0.3395	0	0.2370	0.8378	0.5	0.3370	0.1617

Supplementary Table 3. Sample densities for undoped and Cl doped $PbSnS_2$ crystals.

**Sample, n_H (cm^{-3})**	**Density ($g\ cm^{-3}$)**
$PbSnS_2$, 3.6×10^{11}	6.28
$PbSnS_2$-Cl, 0.8×10^{19}	6.32
$PbSnS_2$-Cl, 1.3×10^{19}	6.32
$PbSnS_2$-Cl, 1.7×10^{19}	6.30

Supplementary References

1. Delaire, O. et al. Phonon density of states and heat capacity of $La_{3-x}Te_4$. *Phys. Rev. B* **80**, 184302 (2009).

2. Zhao, L.-D. et al. High performance thermoelectrics from earth-abundant materials: enhanced figure of merit in PbS by second phase nanostructures. *J. Am. Chem. Soc.* **133**, 20476-20487 (2011).

3. He, W. et al. High thermoelectric performance in low-cost $SnS_{0.91}Se_{0.09}$ crystals. *Science* **365**, 1418-1424 (2019).

4. Zhao, L.-D. et al. Ultrahigh power factor and thermoelectric performance in hole-doped single-crystal SnSe. *Science* **351**, 141-144 (2016).

5. Ravel, B. & Newville, M. ATHENA, ARTEMIS, HEPHAESTUS: data analysis for X-ray absorption spectroscopy using IFEFFIT. *J. Synchrotron Rad.* **12**, 537-541 (2005).

6. Rehr, J. J., Kas, J. J., Vila, F. D., Prangebc, M. P. & Jorissena, K. Parameter-free calculations of X-ray spectra with FEFF9. *Phys. Chem. Chem. Phys.* **12**, 5503-5513 (2010).

7. Kresse, G. & Furthmüller, J. Efficient iterative schemes for *ab initio* total-energy calculations using a plane-wave basis set. *Phys. Rev. B* **54**, 11169-11186 (1996).

8. Kresse, G. & Furthmüller, J. Efficiency of ab-initio total energy calculations for metals and semiconductors using a plane-wave basis set. *Comp. Mater. Sci.* **6**, 15-50 (1996).

9. Perdew, J. P., Burke, K. & Ernzerhof, M. Generalized gradient approximation made simple. *Phys. Rev. Lett.* **77**, 3865-3868 (1996).

10. Blöchl, P. E. Projector augmented-wave method. *Phys. Rev. B* **50**, 17953-17979 (1994).

11. Kresse, G. & Joubert, D. From ultrasoft pseudopotentials to the projector augmented-wave method. *Phys. Rev. B* **59**, 1758-1775 (1999).

12. Monkhorst, H. J. & Pack, J. D. Special points for Brillouin-zone integrations. *Phys. Rev. B* **13**, 5188-5192 (1976).

13. Ong, S. P. et al. Python materials genomics (pymatgen): a robust, open-source python library for materials analysis. *Comp. Mater. Sci.* **68**, 314-319 (2013).

14. Jain, A. et al. Commentary: the materials project: a materials genome approach to accelerating materials innovation. *APL Mater.* **1**, 011002 (2013).

15. Togo, A., Oba, F. & Tanaka, I. First-principles calculations of the ferroelastic transition between rutile-type and $CaCl_2$-type SiO_2 at high pressures. *Phys. Rev. B.* **78**, 134106 (2008).

16. Ioannidou, C., Lioutas, C. B., Frangis, N., Girard, S. N. & Kanatzidis, M. G. Analysis and implications of structural complexity in low lattice thermal conductivity high thermoelectric performance PbTe-$PbSnS_2$ composites. *Chem. Mater.* **28**, 3771-3777 (2016).
